# Chylous ascites following elective abdominal aortic aneurysm repair

**DOI:** 10.1093/jscr/rjaa512

**Published:** 2020-12-30

**Authors:** Sylvie Bowden, Mohammed Firdouse, Graham Roche-Nagle

**Affiliations:** University of Toronto, Department of Medicine, Toronto, Ontario, Canada; University of Toronto, Department of Vascular Surgery, Toronto, Ontario, Canada; Toronto General Hospital, Department of Vascular Surgery, Toronto, Ontario, Canada

## Abstract

Postoperative chylous ascites is a rare complication of abdominal surgery. Chyle depletion results in nutritional, immunologic and metabolic deficiencies, making it a serious and potentially life-threatening condition for which prompt diagnosis and management is imperative. A 72-year-old male was referred for open repair of a 62 cm juxtarenal abdominal aortic aneurysm (AAA). Following resumption of diet, he developed abdominal distention. Therapeutic paracenteses confirmed chylous ascites. Failed conservative management and lymphatic embolization lead to surgical sealance of lymphatic leak using glue. Postoperatively, a full diet was tolerated with no further ascites. Paracentesis is the diagnostic modality of choice in evaluating patients with ascites. Management is challenging and should be multifaceted and tailored to individual patient needs. Cornerstones of therapy include correction of the underlying etiology and conservative measures. When conservative measures fail, other interventions can be considered, such as somatostatin analogs, surgical ligation or glue embolization.

## INTRODUCTION

Postoperative chylous ascites is a rare complication of abdominal surgery caused by disrupted lymphatics. Chyle depletion results in nutritional, immunologic and metabolic deficiencies, making it a potentially life-threatening condition for which prompt diagnosis and management is imperative [1]. Management is challenging and should be based on a stepwise approach, from conservative to surgical [2].

We describe a case of chylous ascites post-elective abdominal aortic aneurysm (AAA) repair, followed by discussion of current trends in management. The associated morbidity makes our paper of interest to vascular and general surgeons. Furthermore, the rarity of this pathology, combined with its associated prolonged hospitalization and increased healthcare expenditure, makes our case of interest to healthcare providers.

## CASE REPORT

A 72-year-old male was referred for a 6 cm juxtarenal AAA. Due to extensive mural thrombus and calcification at the neck, he was not appropriate for endovascular repair. His past medical history includes chronic obstructive pulmonary disease with a 50-pack-year smoking history, chronic kidney disease, hypertension, hyperlipidemia and carotid artery disease requiring endarterectomy.

Intraoperatively, a midline laparotomy was performed to view the aneurysm sac and the neck of the aneurysm was dissected out. As he was very thin, the lymphatics were very visible and divided. The renal vein was also divided and the aortic bifurcation and common iliac arteries were controlled bilaterally. The aneurysm sac was clamped, opened, and a 20 mm Dacron graft was sewn in place. Following completion of the anastomoses, there was excellent hemostasis, strong femoral pulses bilaterally, and the aneurysm sac and abdomen were closed.

**Figure 1:**
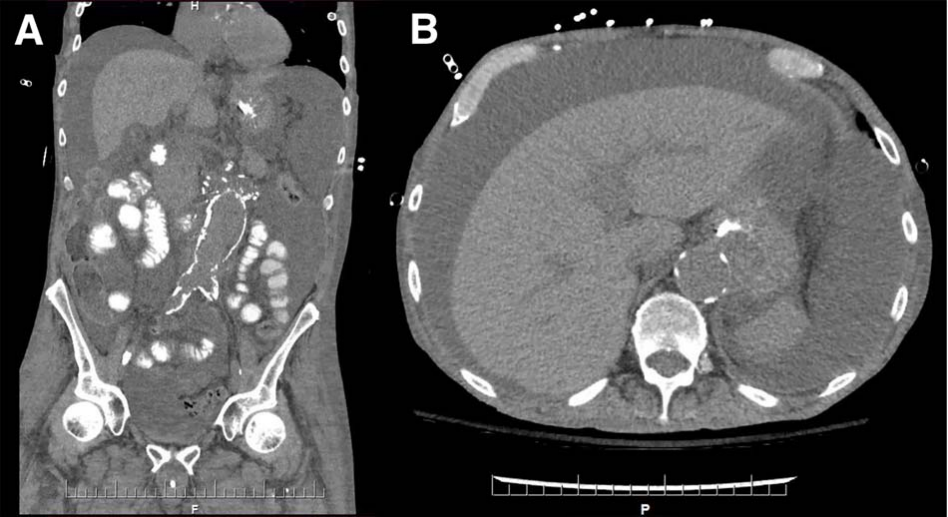
Coronal (**A**) and axial (**B**) sections of non-contrast enhanced abdominal computed tomography showing accumulation of large volume of peritoneal fluid. Aortic graft appears intact.

On postoperative Day 5, diet was resumed and the patient subsequently developed abdominal distention, for which a computer tomography scan was performed (Fig. 1a and b). Therapeutic paracenteses confirmed chylous ascites and he was placed on total parenteral nutrition (TPN) and octreotide. Despite conservative management, the ascites re-accumulated on resumption of oral intake.

**Figure 2:**
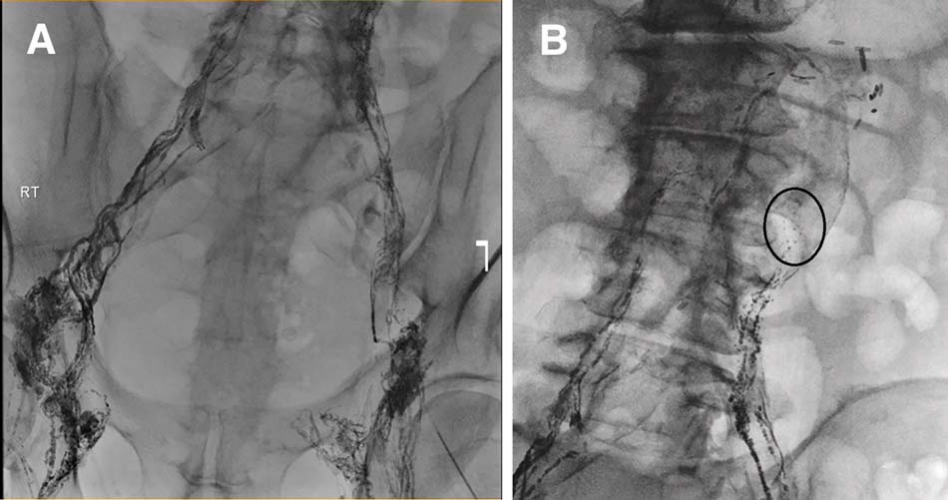
(**A**) Lymphangiogram and unsuccessful attempt at cannulating pelvic lymphatic channels. (**B**) Lymphatic leak is seen at the left of the L3-L4 level.

Failure of conservative management leads to an unsuccessful attempt at lymphatic embolization (Fig. 2a and b). Therefore, the morning of surgery, olive oil was administered orally and lymphatic leakage was identified intraoperatively and sealed using glue. The abdomen was closed with no obvious ongoing lymphatic leak. Full diet was implemented and tolerated with no further ascites.

The patient was transferred to a rehabilitation institution. Unfortunately, he died of ischemic bowel 3 months later.

## DISCUSSION

Chylous ascites is the pathologic accumulation of milky, triglyceride-rich fluid in the peritoneal cavity due to obstruction or trauma of the lymphatics [3]. It has been reported after different surgical procedures with varying incidence; the highest following pancreatic surgery [4]. Numerous vascular procedures may cause trauma to the lymphatics, including AAA repair. While chylous ascites makes up <1% of all abdominal aorta surgical complications, 81% follows AAA repair [5].

Presentation is often insidious, with gradual fluid accumulation causing, in order of frequency, symptoms including, abdominal distention and pain, diarrhea, dysphagia and peripheral edema. Features on physical examination include cachexia, temporal wasting, evidence of pleural effusions or ascites and lower extremity edema [6]. As lymph is rich in nutrients and immunoglobulins, chyle loss results in nutritional, immunologic and metabolic deficiencies, thus making prompt diagnosis and management imperative.

Significant risk factors for chylous ascites include extent of abdominal surgery, early enteral feeding and manipulation of the paraaortic area [4]. Paracentesis is the diagnostic modality of choice in evaluating ascites and a diagnosis of chylous ascites is supported by triglyceride concentration >200 mg/dl and excluded if <50 mg/dl [6]. Diagnosis is further supported through radiographic measures. Lymphangiography and lymphoscintigraphy detect leakage, fistulization and patency of the thoracic duct, as well as select patients for surgery and assess treatment efficacy [6]. Lymphangiography detects leakage location in 75% of cases [4], and is the gold standard diagnostic tool for lymphatic obstruction. While not specific to chylous ascites, computed tomography and magnetic resonance imaging identify intra-abdominal fluid.

The treatment for chylous ascites is multifaceted and should be individualized and based on a stepwise approach, from conservative for up to 2 months to surgical [2]. Cornerstones of therapy include correction of the underlying etiology and conservative measures to optimize nutrition and reduce lymphatic production. The key initial step is a high-protein, medium-chain triglyceride (MCT) diet, with restriction of long-chain triglycerides (LCTs). MCTs bypass the lymphatics and pass directly into the portal system, whereas LCTs enter the lymphatics as chylomicrons [6].

TPN has been used to correct nutritional deficits, allow bowel rest and decrease lymphatic production. However, it is associated with numerous adverse effects, including infection, thrombosis and cholestasis [2], and thus should only be considered if dietary measures fail. Weninger *et al*. [4] found resolution of chylous ascites by conservative management effective in up to 100% of cases, specifically in 77–100% cases with TPN alone, 75% with MCT diet, 100% in MCT diet plus octreotide. Thus, it has been recommended to institute TPN for chyle leakage > 200 ml/day, and MCT for < 200 ml/day or when TPN is contraindicated.

When conservative measures fail, hormonal therapy can be considered. Somatostatin, or its synthetic analog octreotide, decreases intestinal fat absorption and lymph flow [6]. Earlier initiation has shown better outcomes in chylous ascites patients. A study found shorter time to resolution for TPN plus octreotide versus TPN alone [7]. Thus, it is reasonable to consider adding octreotide to TPN, as adverse effects are minor.

If refractory to conservative measures, surgical interventions include ligation or glue embolization of leaking lymphatic channels [6]. An open approach is favored as laparoscopic has been associated with increased lymphatic leakage [2, 3]. Upon visualizating lymphatic leakage, definitive treatment can be achieved using sutures, clips or biologic glue [8]. Lymphangiography and percutaneous glue embolization may also be performed [9]. However, peritoneo-venous shunting is rarely used due to high morbidity rates secondary to infection, shunt obstruction and air embolism [1, 3, 5]. The role and timing for surgical repair remains controversial. Argument for early reintervention includes immediate definitive cessation of chylous leak, thus preventing metabolic and immunologic complications [2]. Argument against includes poor outcomes associated with reoperation in an already malnourished and immunocompromised population.

Overall evidence for management of chylous ascites is sparse and randomized controlled trials are lacking. As such, further research is needed to enhance management.

## INFORMED CONSENT

Written informed consent was obtained from the patient for publication of this case report and accompanying images. A copy of the written consent is available for review by the Editor-in-Chief of this journal on request.
